# circ_0003204 Regulates Cell Growth, Oxidative Stress, and Inflammation in ox-LDL-Induced Vascular Endothelial Cells via Regulating miR-942-5p/HDAC9 Axis

**DOI:** 10.3389/fcvm.2021.646832

**Published:** 2021-04-01

**Authors:** Huan Wan, Ting You, Wei Luo

**Affiliations:** ^1^Department of Cardiology, The First Affiliated Hospital of University of South China, Hengyang, China; ^2^Department of Emergency, The First Affiliated Hospital of University of South China, Hengyang, China

**Keywords:** atherosclerosis, circ_0003204, miR-942-5p, HDAC9, ox-LDL

## Abstract

**Background:** Atherosclerosis (AS) is a typical inflammatory vascular disease. Many reports corroborated that circular RNAs (circRNAs) is involved in AS progression. However, the potential function and possible mechanism of circ_0003204 in AS progression remain indistinct.

**Methods:** Expression level analysis was performed using qRT-PCR and western blot. Cell viability and apoptosis were determined using Cell Counting Kit-8 (CCK-8), flow cytometry, and western blot assays. The status of oxidative stress and inflammation was determined via commercial detection kits and ELISA assay, respectively. The binding relationship was verified via dual-luciferase reporter and RNA immunoprecipitation assays.

**Results:** ox-LDL increased circ_0003204 and HDAC9 levels and decreased miR-942-5p level. Silencing of circ_0003204 enhanced cell viability and inhibited cell apoptosis, oxidative stress and inflammation in ox-LDL-disposed HUVECs. In addition, circ_0003204 targeted miR-942-5p to regulate ox-LDL-resulted HUVECs injury. Also, miR-942-5p affected ox-LDL-triggered HUVECs injury by targeting HDAC9. Furthermore, circ_0003204 elevated HDAC9 expression via decoying miR-942-5p.

**Conclusion:** circ_0003204 aggravated ox-LDL-induced HUVECs damage via modulating miR-942-5p/HDAC9 pathway.

## Background

Atherosclerosis (AS) is a chronic inflammatory disease marked by atherosclerotic plaque (1), posing a serious threat to the cardiovascular system. Endothelial cells (ECs) and vascular smooth muscle cells (VSMCs) are important cells that regulate the progression of AS (2). The pathogenesis of AS includes increased low-density lipoprotein oxidation, ECs dysfunction and apoptosis caused by mitochondrial dysfunction (3). Human umbilical vein ECs (HUVECs) are typical cells used to study AS (4). Therefore, exploring the potential mechanism of oxidized low-density lipoprotein (ox-LDL)-mediated HUVECs injury is crucial to understanding the pathogenesis of AS.

Circular RNAs (circRNAs) are neotype RNA molecules without 5′ to 3′ polarity produced by back-splicing (5). Mounting studies have corroborated that circRNAs are closely related to cardiovascular diseases (6), including AS. For example, down-regulation of circ_0029589 suppressed the growth and motility of ox-LDL-mediated VSMCs in AS via competitively combining with miR-424-5p to regulate IGF2 (7). Qin et al. (8) suggested that depletion of circ_0003645 mitigated ox-LDL-mediated apoptosis and inflammation in HUVECs via regulating NF-κB pathway. In addition, circ_0003204 was prominently increased in ox-LDL-disposed human aorta endothelial cells (HAECs), and up-regulation of circ_0003204 hindered HAECs growth and migration in AS (9). Also, Liu *et al*. revealed that circ_0003204 silencing accelerated ox-LDL-triggered proliferation and angiogenesis in HUVECs (10). Nonetheless, the exact function and mechanism of circ_0003204 in endothelial injury have not been elucidated.

Substantial literatures have demonstrated that circRNAs participate in the development of cardiovascular diseases via serving as microRNA (miRNA) sponges (11). Additionally, miRNAs contribute to target gene silencing and degradation via base-pairing with mRNA 3′UTR (12, 13). Moreover, increasing reports have verified that aberrantly expressed miRNAs exert crucial regulatory effects on many biological processes of AS (14, 15). For instance, miR-151 restrained the apoptosis of endothelial cells in ox-LDL-induced HAECs via repressing Interleukin-17A (16). Qin et al. (17) found that miR-328-3p ameliorated ox-LDL-resulted HUVECs injury in AS by binding to FOXO4. Besides, we predicted that circ_0003204 might target miR-942-5p through bioinformatics analysis. Also, Hua et al. (18) unveiled that miR-942 down-regulation overturned the inhibition of ZEB1-AS1 silencing on ox-LDL-induced endothelial injury.

Herein, we established an ox-LDL-disposed HUVECs model and investigated the expression pattern and biological function of circ_0003204 in ox-LDL-stimulated HUVECs. Furthermore, we explored the interaction between circ_0003204 and miR-942-5p/histone deacetylase 9 (HDAC9) pathway in ox-LDL-mediated HUVECs.

## Materials and Methods

### Cell Culture

Human umbilical vein endothelial cells (HUVECs) were commercially acquired from American Type Culture Collection (cat. no. CRL-1730; ATCC, Manassas, VA, USA) and cultured in F-12K Medium (Youkang Biotech, Beijing, China) supplemented with 10% fetal bovine serum (FBS; Youkang Biotech) with 5% CO_2_ at 37°C. Additionally, HUVECs were stimulated with 100 mg/L ox-LDL (Solarbio, Beijing, China) for 48 h to construct an *in vivo* AS model.

### Cell Transfection

circ_0003204 small interfering RNA (si-circ_0003204#1, si-circ_0003204#2 and si-circ_0003204#3) and negative control (si-NC), miR-942-5p mimic and the control (miRNA NC), HDAC9 overexpression vector (pc-HDAC9) and negative control (pc-NC), miR-942-5p inhibitor and the control (inhibitor NC) were synthesized by Genechem (Shanghai, China). HUVECs transfection was conducted using Lipofectamine 3000 (Invitrogen, Carlsbad, CA, USA) when cells reached ~80% confluence.

### Quantitative Real-Time PCR (qRT-PCR)

TRIzol reagent (Leagene, Beijing, China) was applied for extracting total RNA. Afterwards, cDNA was synthesized using the specific reverse transcription kit (Vazyme, Nanjing, China). For detecting RNA levels, qRT-PCR reactions were carried out using SYBR Green Master Mix (Vazyme). RNA levels were quantified via the 2^−ΔΔCt^ method. GAPDH (for circ_0003204 and HDAC9) and U6 (for miR-942-5p) were regarded endogenous controls. The primers included: circ_0003204-F: 5′-C A T G G G G C T G T G T C A C C T G-3′, circ_0003204-R: 5′-G G C A A C T G G T G T G G A A G A G A-3′; miR-942-5p-F: 5′-C T T C T C T G T T T T G G C C A T G T G-3′, miR-942-5p-R: 5′-C T C T A C A G C T A T A T T G C C A G C C A C-3′; HDAC9-F: 5′-A G T A G A G A G G C A T C G C A G A G A-3′, HDAC9-R: 5′-G G A G T G T C T T T C G T T G C T G A T-3′; GAPDH-F: 5′-G C T G A G T A C G T C G T G G A G T C-3′, GAPDH-R: 5′-A G T T G G T G G T G C A G G A G G C-3′; U6-F: 5′-C T C G C T T C G G C A G C A C A-3′, U6-R: 5′-A A C G C T T C A C G A A T T T G C G T-3′.

### Cell Viability Assay

2 × 10^3^ HUVECs were added into 96-well plates and then exposed to ox-LDL for the indicated time. Afterwards, the cells were incubated with 10 μL CCK-8 reagent (Boster, Wuhan, China) for 4 h. Finally, cell viability was assessed by measuring the absorbance at 450 nm using a microplate reader (Bio-Rad, Hercules, CA, USA).

### Flow Cytometry

Cell apoptosis was evaluated by adopting AnnexinV-FITC/Propidium Iodide (PI) Apoptosis Detection kit (Abcam, Cambridge, UK). HUVECs in 6-well plates were resuspended in binding buffer and then stained with AnnexinV-FITC and PI. Finally, the apoptosis analysis was conducted using flow cytometer (Beckman Coulter, Miami, FL, USA).

### Western Blot Assay

After extracting protein using RIPA buffer (Beyotime, Shanghai, China), the protein was quantified using BCA Protein Assay Kit (Tiangen, Beijing, China). Then, the protein samples were separated by 10% SDS-PAGE and transferred to PVDF membranes (Beyotime). Following blocking with 5% skimmed milk for 2 h at room temperature, the membranes interacted with primary antibodies against proliferating cell nuclear antigen (PCNA; 1:1000, ab18197, Abcam), Bcl-2 associated X protein (Bax; 1:1000, ab104156, Abcam), HDAC9 (1:20000, ab109446, Abcam), or GAPDH (1:2500, ab9485, Abcam) overnight at 4°C. After washing, the membranes were probed with HRP-coupled secondary antibody (1:25000, ab205718, Abcam) at room temperature for 2 h. Finally, the signal intensity was measured using ECL reagent (Absin, Shanghai, China).

### Measurement of MDA, SOD, and ROS

HUVECs treated with different conditions were harvested and lysed, and the supernatant was collected by centrifugation at 12,000 g for 5 min. Afterwards, malondialdehyde (MDA) level, superoxide dismutase (SOD) activity and reactive oxygen species (ROS) formation were determined using the corresponding kits (Abcam) according to the manufacturer's requirements.

### Enzyme Linked Immunosorbent Assay (ELISA)

Transfected HUVECs were stimulated with ox-LDL for 48 h, and the culture medium were collected. Subsequently, the levels of interleukin 1 beta (IL-1β), interleukin 6 (IL-6) and tumor necrosis factor alpha (TNF-α) in HUVECs culture medium were examined using the specific ELISA kits (Boster) following the manufacturer's instructions.

### Dual-Luciferase Reporter Assay

The fragment of circ_0003204 or HDAC9 3′UTR containing wild-type or mutant miR-942-5p binding site was inserted into pmirGLO vector (LMAI Bio, Shanghai, China) to form WT-circ_0003204, MUT-circ_0003204, WT-HDAC9-3′UTR, or MUT-HDAC9-3′UTR reporter. Subsequently, the constructed vector and miRNA NC or miR-942-5p mimic were introduced into HUVECs. The relative luciferase activities were tested via Dual-Lucy Assay Kit (Solarbio).

### RNA Immunoprecipitation (RIP) Assay

RIP analysis was implemented using EZ-Magna RIP kit (Millipore, Billerica, MA, USA). After lysing cells with RIP lysis buffer, cell lysates were incubated with magnetic beads combined with anti-Ago2 or anti-IgG (as the control). Additionally, qRT-PCR analysis was utilized to measure the abundance of circ_0003204 and miR-942-5p.

### Statistical Analysis

Data were expressed as mean ± standard deviation (SD) in three independent replicates by using GraphPad Prism 7 software (GraphPad, San Diego, CA, USA). Student's *t*-test or one-way analysis of variance was utilized to evaluate the differences between groups. *P* < 0.05 was considered statistically significant.

## Results

### circ_0003204 Expression Is Increased by ox-LDL in HUVECs

**Figure 1:**
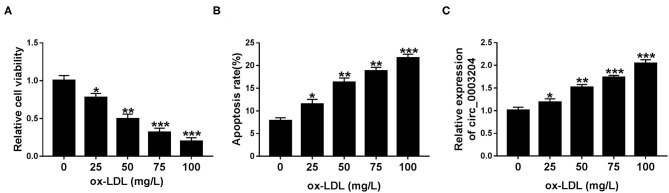
Expression of circ_0003204 in ox-LDL-disposed HUVECs. **(A–C)** HUVECs were stimulated by increasing doses of ox-LDL for 48 h. **(A,B)** HUVECs viability and apoptosis were determined via CCK-8 and flow cytometry. **(C)** qRT-PCR analysis was utilized to measure circ_0003204 level in HUVECs stimulated with different doses of ox-LDL. Data were presented as mean ± SD, *n* = 3. *P < 0.05, **P < 0.01, and ***P < 0.001.

To explore the effect of ox-LDL on HUVECs, the cytotoxicity and apoptosis rate were tested in HUVECs exposed to different concentrations of ox-LDL (0, 25, 50, 75, and 100 mg/L). CCK-8 and flow cytometry assays showed that ox-LDL strikingly reduced the viability of HUVECs and increased the apoptosis rate of HUVECs in a dose-dependent manner (Figures 1A,B). Furthermore, we also examined the effect of ox-LDL at different doses on circ_0003204 expression in HUVECs. As depicted in Figure 1C, ox-LDL significantly increased the expression of circ_0003204 in a concentration-dependent manner. Therefore, the concentration of 100 mg/L was selected for subsequent experiments.

### Knockdown of circ_0003204 Enhances Cell Viability and Inhibits Cell Apoptosis, Oxidative Stress and Inflammation in ox-LDL-Disposed HUVECs

**Figure 2:**
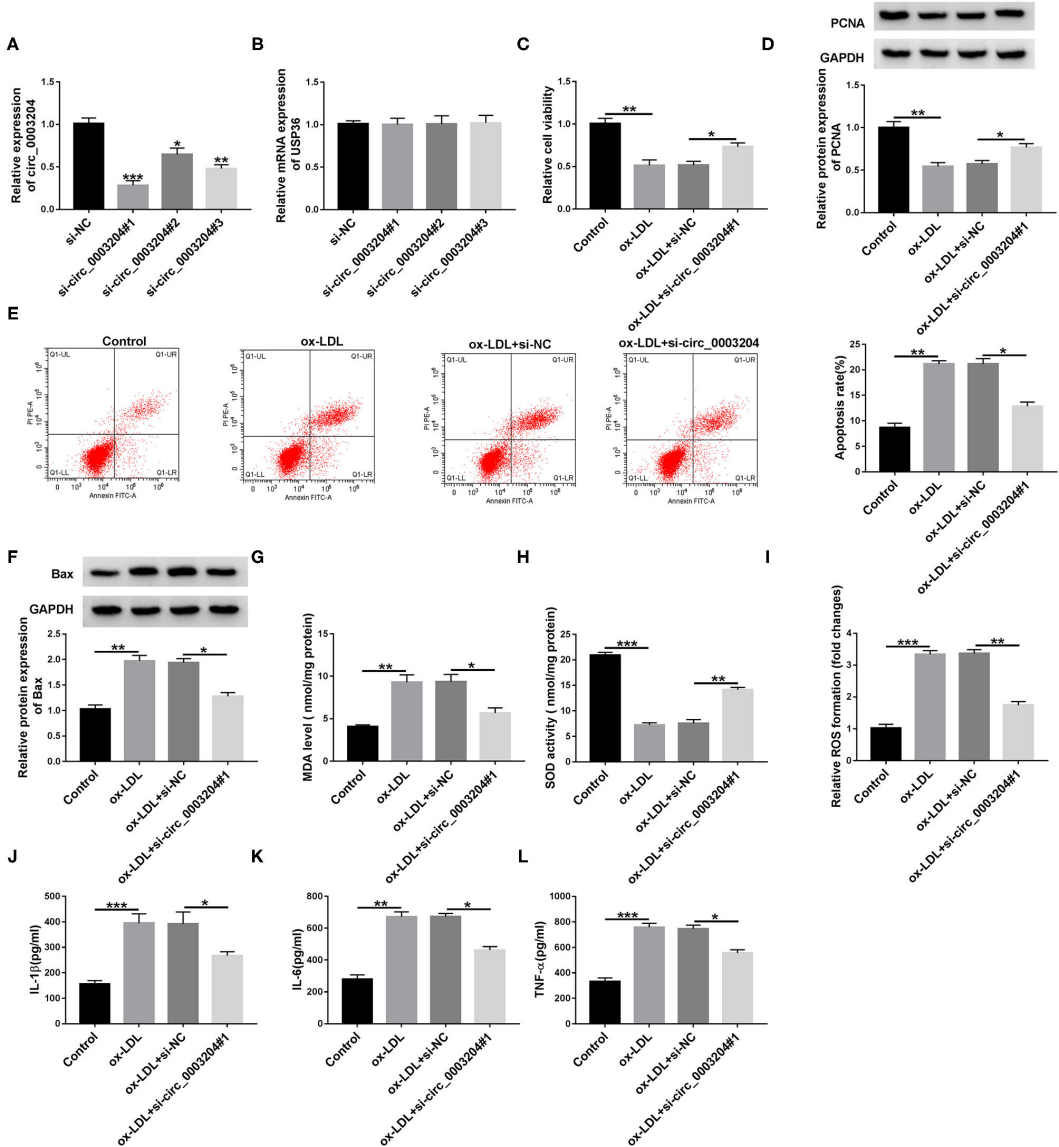
Effect of circ_0003204 silence on ox-LDL-triggered HUVECs injury in HUVECs. **(A,B)** HUVECs were introduced with circ_0003204 siRNA or si-NC. circ_0003204 and USP36 levels were measured by qRT-PCR. **(C-L)** Transfected HUVECs were stimulated with 100 mg/L ox-LDL. **(C,D)** Cell viability and PCNA level were tested using CCK-8 and western blot assays, respectively. **(E,F)** Cell apoptosis and Bax level were measured using flow cytometry and western blot assays, respectively. **(G–I)** MDA level, SOD activity and ROS formation were measured using commercial kits. **(J–L)** The levels of inflammatory cytokines were detected via ELISA. Data were presented as mean ± SD, *n* = 3. *P < 0.05, **P < 0.01, and ***P < 0.001.

First of all, qRT-PCR analysis showed that transfection of circ_0003204 siRNA remarkably reduced circ_0003204 expression, but had no effect on linear USP36 (Figures 2A,B). In addition, si-circ_0003204#1 with the most significant knockdown efficiency was selected for subsequent experiments. To investigate the role of circ_0003204 in ox-LDL-mediated damage, HUVECs transduced with si-NC or si-circ_0003204#1 were stimulated with 100 mg/ml ox-LDL. CCK-8 analysis showed that circ_0003204 silencing and ox-LDL stimulation strikingly increased the viability of HUVECs compared with ox-LDL treatment alone (Figure 2C). In addition, ox-LDL-mediated down-regulation of PCNA expression was restored by circ_0003204 depletion (Figure 2D and Supplementary Figure 3A). Flow cytometry suggested that down-regulation of circ_0003204 significantly decelerated ox-LDL-triggered apoptosis in HUVECs (Figure 2E). In addition, the Bcl-2 family composed of pro-apoptotic (such as Bax) and anti-apoptotic (such as Bcl-2) members plays an important role in cell death (19). As expected, circ_0003204 knockdown markedly decreased Bax expression and increased Bcl-2 expression in ox-LDL-treated HUVECs (Figure 2F, Supplementary Figures 2A, 3B,C). Moreover, ox-LDL increased MDA level and ROS formation and decreased SOD activity, while these changes were reversed by suppressing circ_0003204 (Figures 2G–I). Besides, ELISA revealed that circ_0003204 silence attenuated the increase of IL-1β, IL-6, and TNF-α caused by ox-LDL stimulation in HUVECs (Figures 2J–L). Overall, these data indicated that silencing of circ_0003204 elevated cell viability and hindered cell apoptosis, oxidative stress and inflammation in ox-LDL-stimulated HUVECs.

### circ_0003204 Directly Targets miR-942-5p

**Figure 3:**
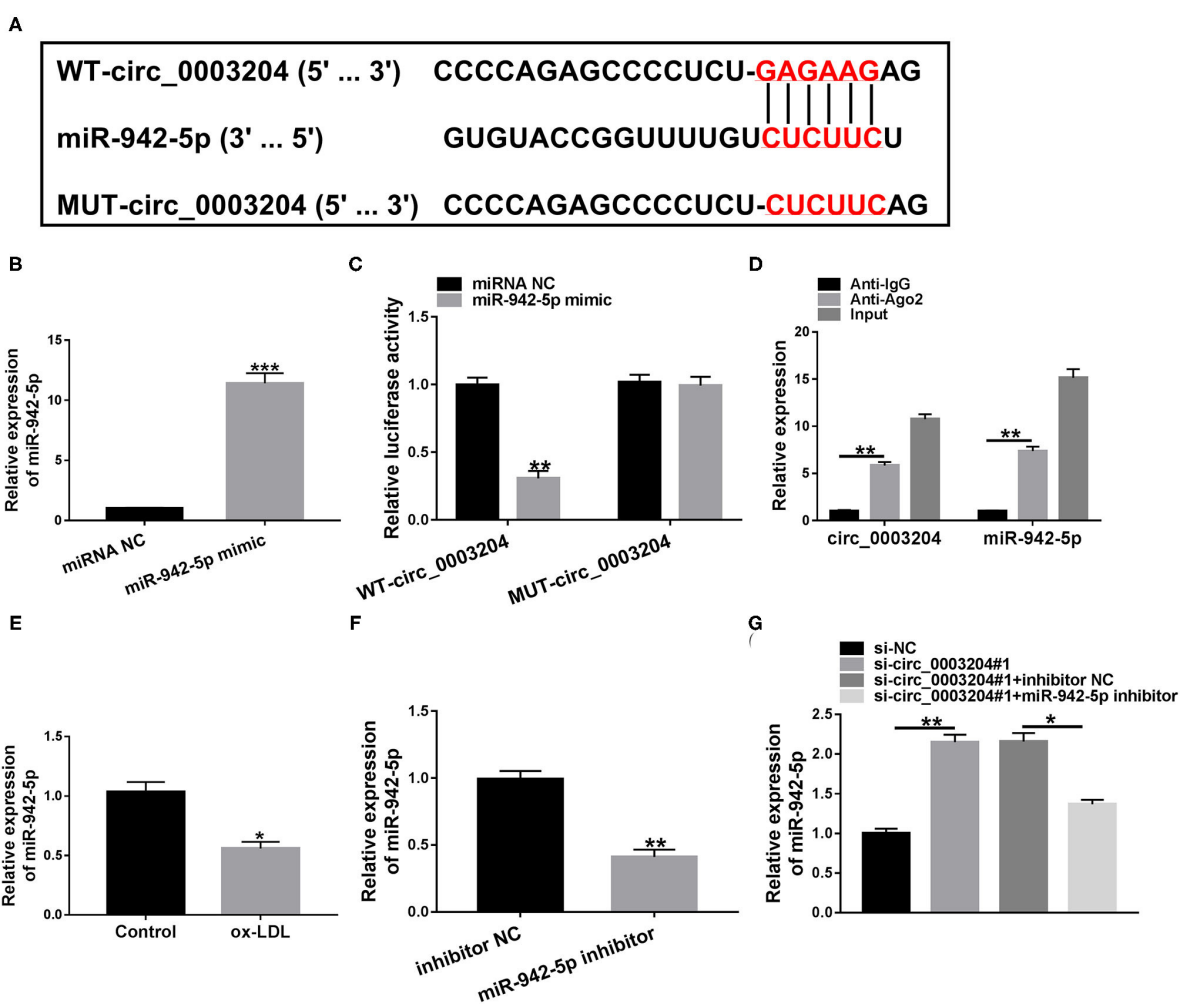
circ_0003204 directly targets miR-942-5p. **(A)** The putative binding site between circ_0003204 and miR-942-5p was described by Circular RNA Interactome. **(B)** The transfection efficiency of miR-942-5p mimic was determined by qRT-PCR. **(C,D)** Dual-luciferase reporter and RIP assays were applied to validate the targeting relationship between circ_0003204 and miR-942-5p. **(E)** miR-942-5p level was detected in HUVECs under ox-LDL stimulation. **(F)** The knockdown efficiency of miR-942-5p inhibitor was validated by qRT-PCR. **(G)** miR-942-5p level was measured in HUVECs transduced with si-circ_0003204#1 or/and miR-942-5p inhibitor. Data were presented as mean ± SD, *n* = 3. *P < 0.05, **P < 0.01, and ***P < 0.001.

Circular RNA Interactome predicted that circ_0003204 might sponged miR-942-5p (Figure 3A). As illustrated in Figure 3B, miR-942-5p mimic remarkably elevated miR-942-5p level in HUVECs. To illuminate the relationship between circ_0003204 and miR-942-5p, dual-luciferase reporter and RIP assays were applied. The results identified that miR-942-5p overexpression markedly declined the luciferase activity of WT-circ_0003204 reporter (Figure 3C). RIP analysis showed that circ_0003204 and miR-942-5p were prominently enriched in the anti-Ago2 group relative to the anti-lgG group (Figure 3D). Additionally, ox-LDL inhibited the expression of miR-942-5p compared with the control group (Figure 3E). As depicted in Figure 3F, miR-942-5p inhibitor had a marked knockdown efficiency. Furthermore, introduction of miR-942-5p inhibitor abolished the elevation in miR-942-5p level caused by circ_0003204 depletion (Figure 3G). These data indicated that circ_0003204 directly sponged and negatively regulated miR-942-5p in HUVECs.

### circ_0003204 Silencing Alleviates ox-LDL-Resulted HUVECs Injury by Regulating miR-942-5p

**Figure 4:**
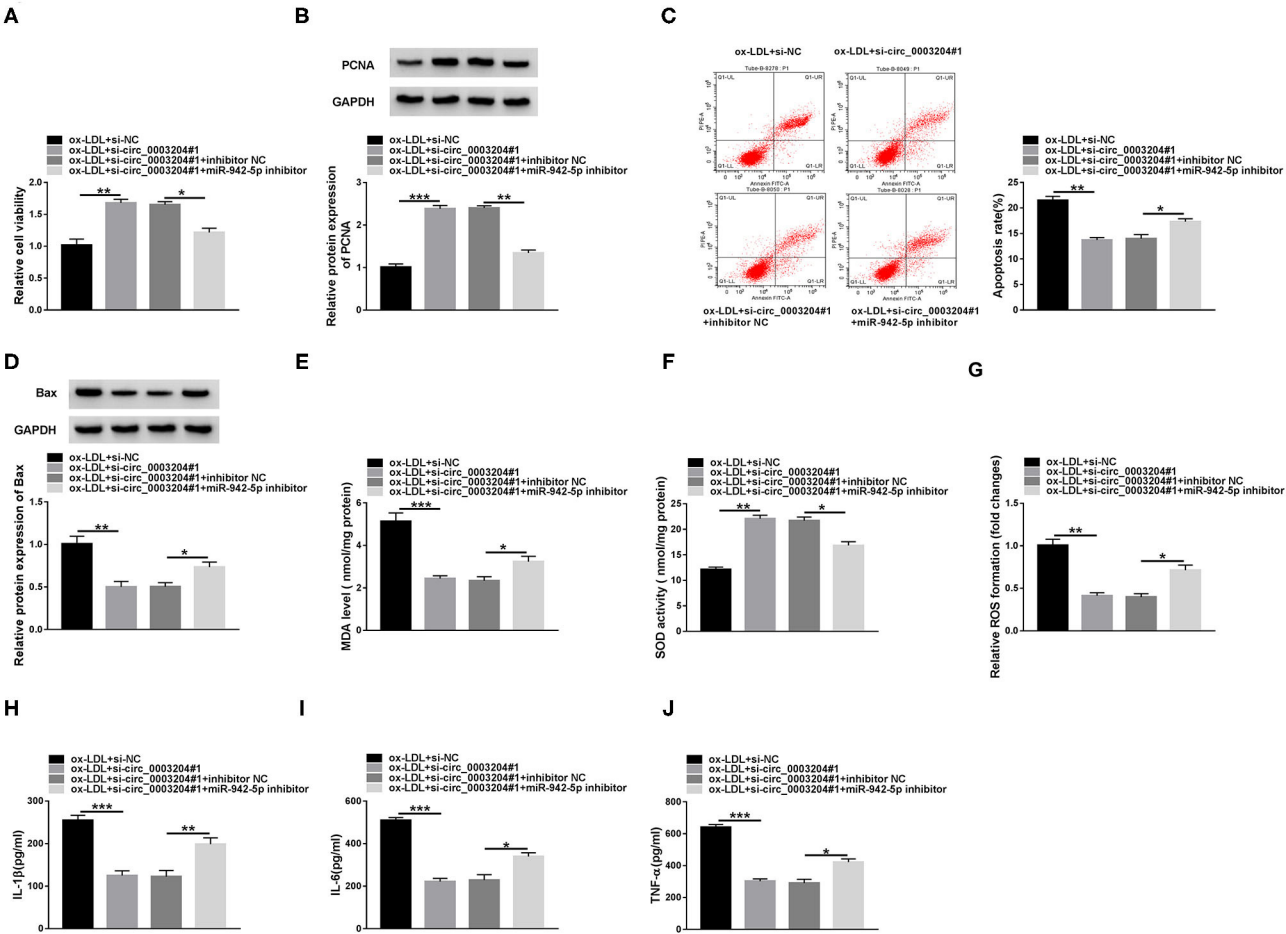
circ_0003204 silencing alleviates ox-LDL-resulted HUVECs injury by regulating miR-942-5p. HUVECs were introduced with si-circ_0003204#1 or/and miR-942-5p inhibitor, followed by treatment with 100 mg/L ox-LDL. Cell viability **(A)**, apoptosis **(C)**, and the levels of PCNA and Bax **(B,D)** were assessed via CCK-8, flow cytometry and western blot assays. **(E-G)** MDA level, SOD activity and ROS formation were examined by commercial kits. **(H-J)** The levels of inflammatory cytokines were measured by ELISA. Data were presented as mean ± SD, *n* = 3. *P < 0.05, **P < 0.01, and ***P < 0.001.

To explore whether circ_0003204 targeted miR-942-5p to regulate HUVECs injury, the transfected HUVECs were stimulated with 100 mg/L ox-LDL. CCK-8, western blot and qRT-PCR assays showed that circ_0003204 depletion enhanced the viability of ox-LDL-disposed HUVECs, while the impact was partially reversed by down-regulating miR-942-5p (Figures 4A,B and Supplementary Figure 3D). Flow cytometry, western blot and qRT-PCR suggested that circ_0003204 silencing suppressed HUVECs apoptosis under ox-LDL treatment, whereas co-transfection of miR-942-5p inhibitor partially abolished this impact (Figures 4C,D, Supplementary Figures 2B, 3E,F). In addition, circ_0003204 knockdown impeded oxidative stress in ox-LDL-disposed HUVECs by reducing MDA and ROS and increasing SOD activity, while this impact was partially mitigated by repressing miR-942-5p (Figures 4E–G). Besides, knockdown of miR-942-5p partially eliminated the inhibition of circ_0003204 silence on inflammation (Figures 4H–J). These data identified that circ_0003204 worsened ox-LDL-resulted HUVECs injury via modulating miR-942-5p.

### miR-942-5p Directly Targets HDAC9

**Figure 5:**
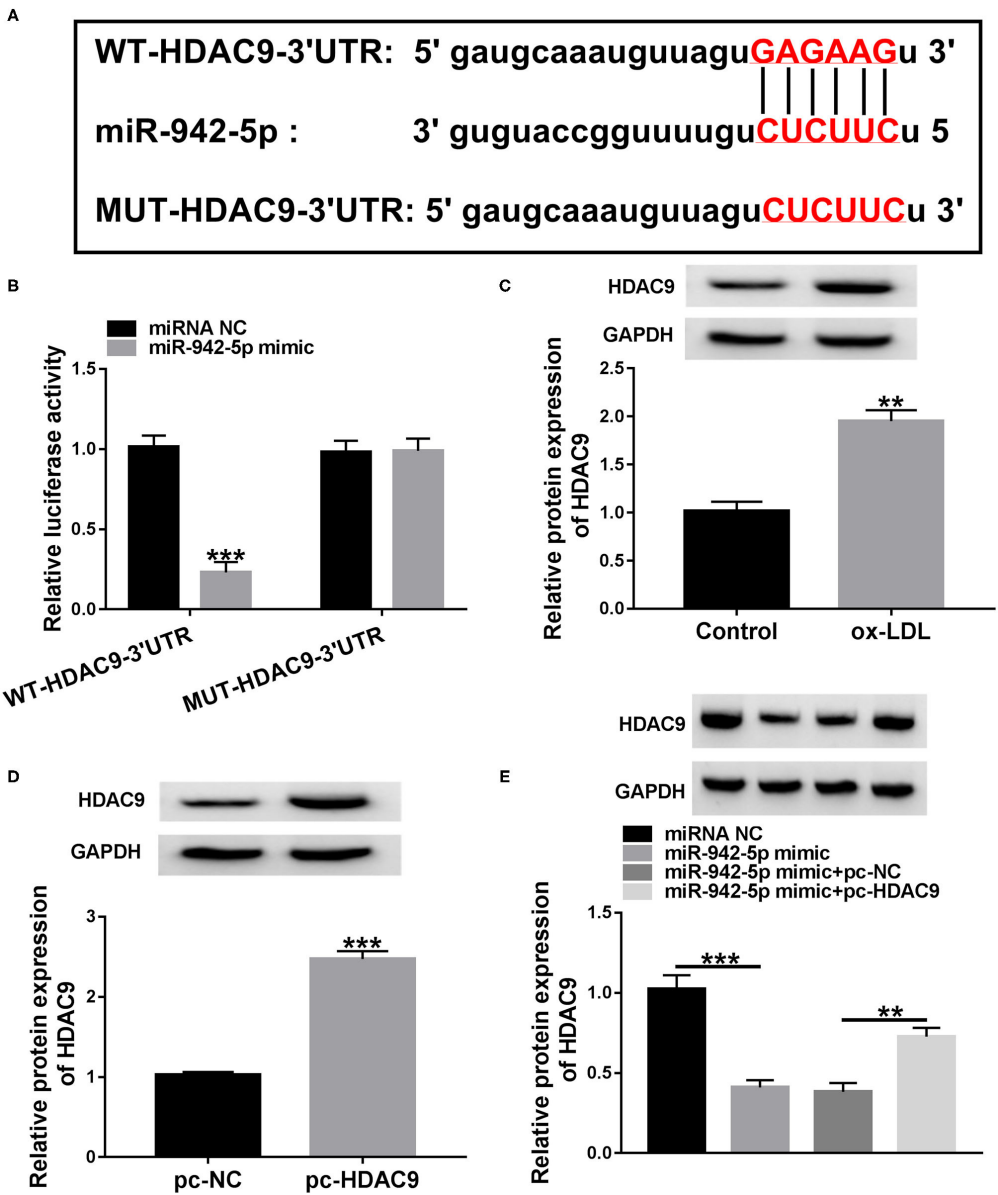
miR-942-5p directly targets HDAC9. **(A)** The predicted binding site of miR-942-5p in HDAC9 3′UTR was displayed by starBase. **(B)** The targeting relationship was validated using dual-luciferase reporter analysis. **(C)** HDAC9 protein level was measured in ox-LDL-disposed HUVECs. **(D)** The overexpression efficiency of pc-HDAC9 was tested using western blot. **(E)** HDAC9 protein level was detected in HUVECs transduced with miR-942-5p mimic or/and pc-HDAC9. Data were presented as mean ± SD, *n* = 3. **P < 0.01, and ***P < 0.001.

Next, we predicted the possible target genes of miR-942-5p through starBase database. We predicted 6 candidate genes (PANK1, TRIB1, BMPR2, HDAC9, THBS1, and LARP1) that might bind to miR-942-5p and played a role in cardiovascular disease. Next, qRT-PCR was used to detect the expression levels of 6 candidate genes after miR-942-5p knockdown. The results showed that the up-regulation of HDAC9 was the most significant, so we chose HDAC9 as a possible target of miR-942-5p for follow-up studies (Supplementary Figure 1). As displayed in Figure 5A, miR-942-5p and HDAC9 3′UTR possessed a putative binding site. Additionally, dual-luciferase reporter analysis suggested that miR-942-5p mimic significantly declined the luciferase activity of WT-HDAC9-3′UTR reporter (Figure 5B). Besides, ox-LDL remarkably increased the protein and mRNA expression of HDAC9 relative to the control group (Figure 5C and Supplementary Figure 4A). Western blot and qRT-PCR analysis exhibited that HDAC9 protein and mRNA levels in the pc-HDAC9 group were markedly elevated compared with the pc-NC group (Figure 5D and Supplementary Figure 4B). Furthermore, co-transfection with miR-942-5p mimic and pc-HDAC9 abrogated the reduction in HDAC9 protein and mRNA levels caused by miR-942-5p overexpression (Figure 5E and Supplementary Figure 4C). These results evidenced that miR-942-5p directly targeted and negatively regulated HDAC9.

### miR-942-5p Attenuates ox-LDL-Resulted HUVECs Injuryi *Via* Targeting HDAC9

**Figure 6:**
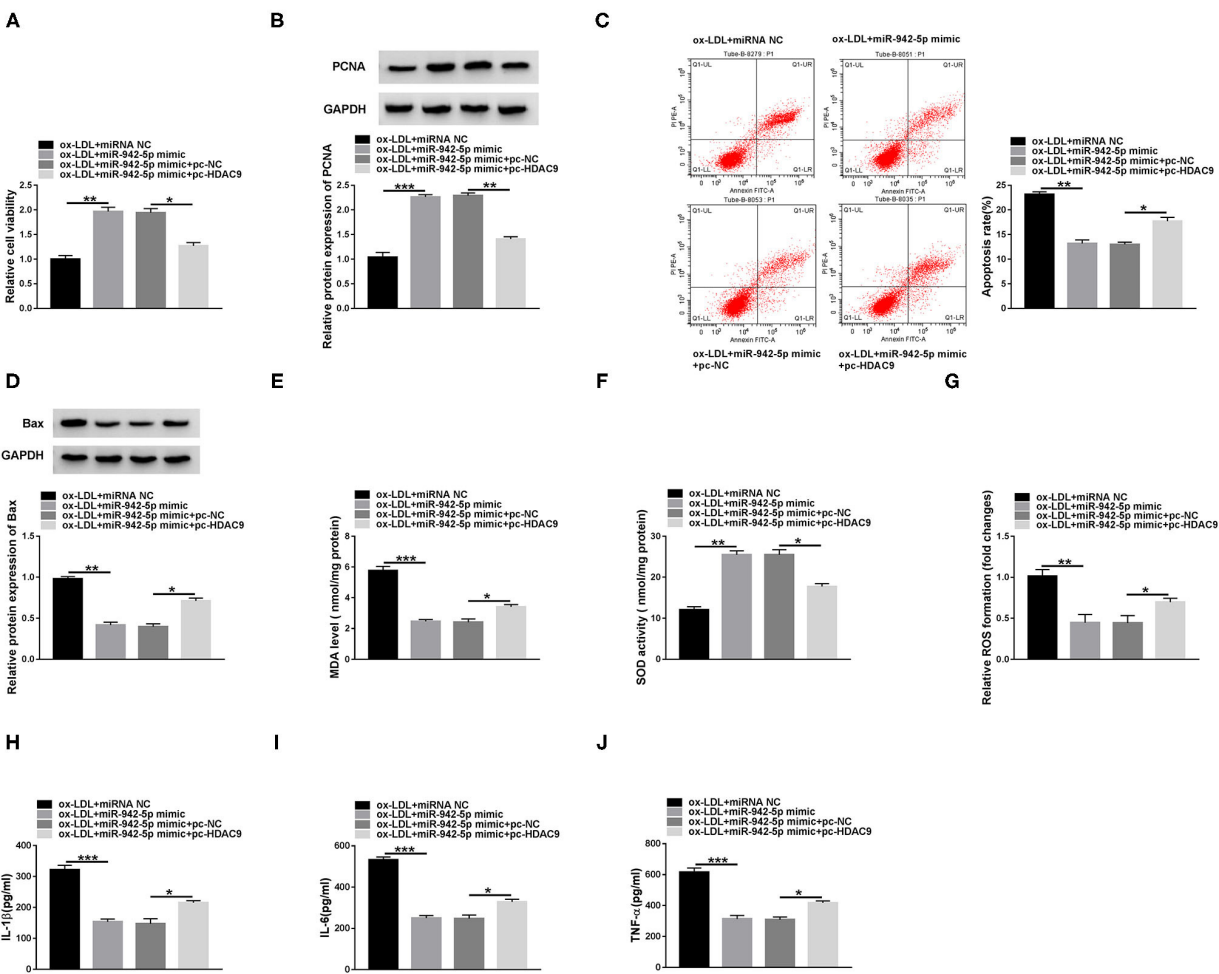
miR-942-5p attenuates ox-LDL-resulted HUVECs injury via targeting HDAC9. HUVECs were introduced with miR-942-5p mimic or/and pc-HDAC9 and then stimulated with 100 mg/L ox-LDL. Cell viability **(A)**, apoptosis **(C)**, and the levels of PCNA and Bax **(B,D)** were evaluated by CCK-8 assay, flow cytometry and western blot. **(E-G)** MDA level, SOD activity and ROS formation were detected by commercial kits. **(H-J)** The levels of inflammatory cytokines were examined using ELISA. Data were presented as mean ± SD, *n* = 3. *P < 0.05, **P < 0.01, and ***P < 0.001.

To clarify whether miR-942-5p mediated HDAC9 to affect HUVECs injury, HUVECs were transfected with miR-942-5p mimic or/and pc-HDAC9, and then exposed to 100 mg/L ox-LDL. Rescue experiments confirmed that miR-942-5p up-regulation increased the viability of ox-LDL-disposed HUVECs, while co-transfection of miR-942-5p mimic and pc-HDAC9 abolished this impact (Figures 6A,B and Supplementary Figure 3G). In addition, miR-942-5p mimic-resulted inhibitory effect on apoptosis was partially abrogated by up-regulating HDAC9 (Figures 6C,D, Supplementary Figures 2C, 3H,I). Moreover, miR-942-5p mimic suppressed oxidative stress in ox-LDL-stimulated HUVECs, which was overturned by overexpressing HDAC9 (Figures 6E–G). Furthermore, miR-942-5p up-regulation-induced inhibitory effect on inflammation was partially abolished after transfection with pc-HDAC9 (Figures 6H–J). These data indicated that miR-942-5p alleviated ox-LDL-mediated HUVECs damage via inhibiting HDAC9.

### circ_0003204 Regulates HDAC9 Expression via Sponging miR-942-5p

**Figure 7:**
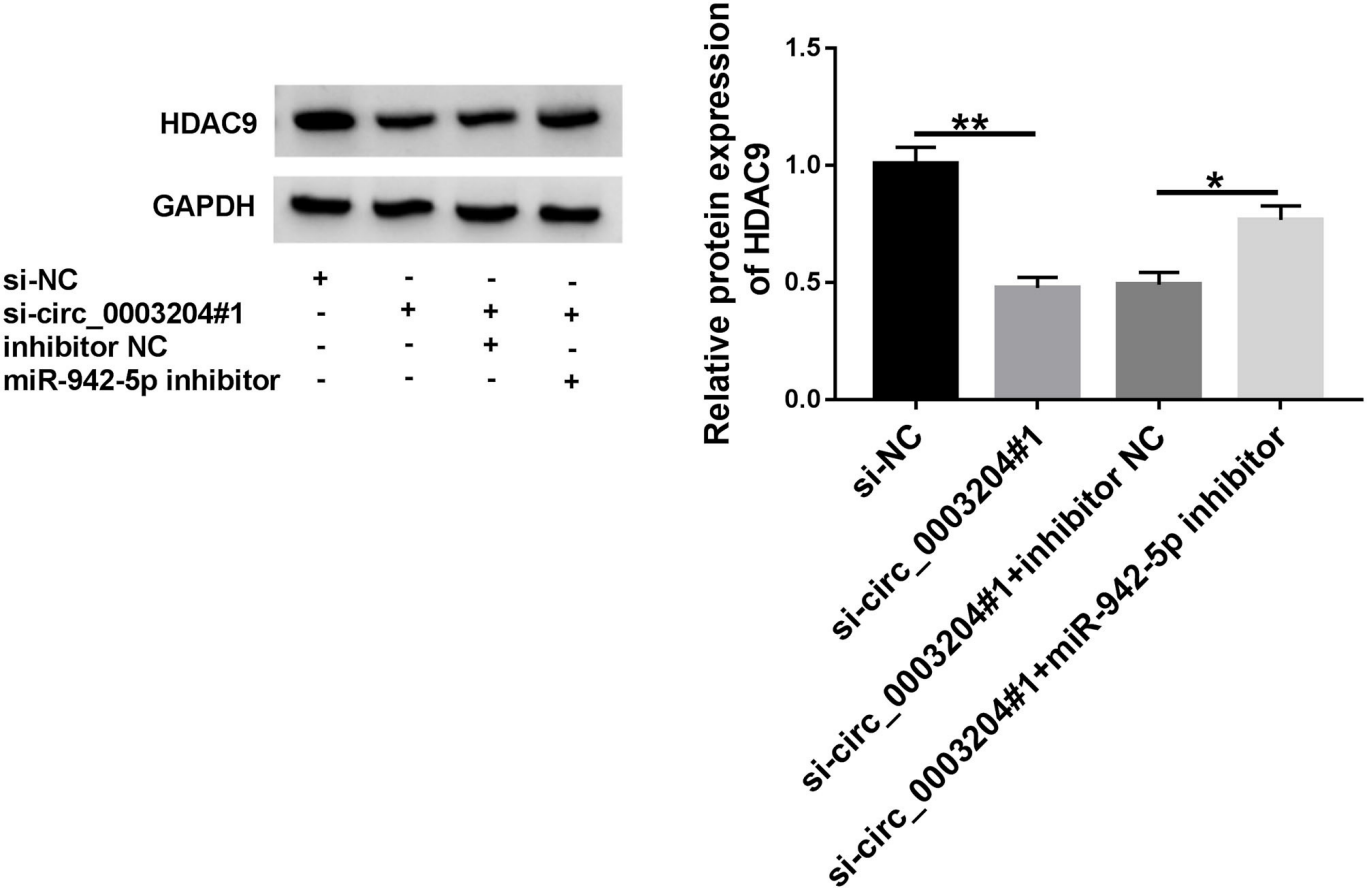
circ_0003204 regulates HDAC9 expression via sponging miR-942-5p. HUVECs were introduced with si-circ_0003204#1 or/and miR-942-5p inhibitor, and HDAC9 expression was tested using western blot. Data were presented as mean ± SD, *n* = 3. *P < 0.05, and **P < 0.01.

To investigate the interaction between HDAC9 and circ_0003204/miR-942-5p axis, HDAC9 expression was examined in HUVECs transfected with si-circ_0003204#1 or/and miR-942-5p inhibitor. Western blot and qRT-PCR assays showed that knockdown of miR-942-5p reversed the decrease in HDAC9 protein and mRNA levels caused by circ_0003204 interference (Figure 7 and Supplementary Figure 4C). These data indicated that circ_0003204 sponged miR-942-5p to elevate HDAC9 expression.

## Discussion

Mounting evidence has corroborated that endothelial dysfunction induces oxidative stress and inflammation, thereby promoting the development of AS (20, 21). In addition, ox-LDL contributes to endothelial cell dysfunction and damage, as well as VSMCs growth and migration (22, 23). Therefore, AS cell model can be established by stimulating HUVECs with ox-LDL. Besides, substantial investigations have demonstrated that circRNAs are recognized as critical mediators in AS progression (24). In addition, the cAMP, AMPK, and FOXO signaling pathways have been verified to be involved in the progression of atherosclerosis (25–27). In the current research, we unveiled that the new regulatory axis of circ_0003204/miR-942-5p/HDAC9 might provide a promising therapeutic target for AS.

Herein, we validated that circ_0003204 was conspicuously elevated in ox-LDL-treated HUVECs. Furthermore, circ_0003204 expedited HUVECs injury by inhibiting cell proliferation and inducing oxidative stress and inflammation under ox-LDL stimulation. In terms of mechanism, plentiful studies have manifested that circRNAs regulate various biological functions in AS via participating in competing endogenous RNA (ceRNA) network (28). For instance, circ_0010283 facilitated the growth and migration of ox-LDL-stimulated VMSCs through sponging miR-370-3p and elevating HMGB1 expression (29). Circ_0124644 aggravated endothelial injury triggered by ox-LDL stimulation in HUVECs via absorbing miR-149-5p to activate PAPP-A (30). Circ_CHFR facilitated ox-LDL-resulted cell growth, migration and inflammation in VSMCs by decoying miR-214-3p (31). Additionally, Zheng et al. (9) discovered that circ_0003204 restrained endothelial cell proliferation, migration and angiogenesis by sequestering miR-370-3p to regulate TGFβR2/phosph-SMAD3 signaling. However, the underlying mechanism of circ_0003204 in AS needs further investigation, and the purpose of this study is to discover a new circRNA-miRNA-mRNA axis involved in the pathogenesis of AS.

Hence, we investigated the potential ceRNA mechanism of circ_0003204 in HUVECs through bioinformatics prediction and experimental analysis. Based on previous research, miR-942-5p was selected as a potential target for circ_0003204. Several studies have corroborated that miR-942-5p plays a pro-oncogenic role in different cancers, including gastric carcinoma (32), cervical cancer (33), and lung cancer (34). A recent research demonstrated that ox-LDL treatment strikingly down-regulated miR-942 in endothelial cells and macrophages (18). Herein, we disclosed that miR-942-5p down-regulation abrogated the impact of circ_0003204 depletion on ox-LDL-triggered HUVECs damage.

Moreover, accumulating investigations have elaborated that circRNAs participate in many biological processes by indirectly regulating gene expression via competing with mRNA 3′UTR for miRNA binding sites (35). Our report indicated that miR-942-5p could target HDAC9 by combining with its 3′UTR. HDAC9 belongs to the class IIa histone deacetylases, which has a regulatory effect on the cardiovascular, musculoskeletal, nervous, and immune systems (36, 37). The absence of HDAC9 hinders AS progression by reducing inflammation and reversing cholesterol transport (38). Malhotra et al. (39) reported that HDAC9 depletion blocked aortic calcification and increased VSMCs contractility. Han et al. (40) indicated that HDAC9 was overtly increased in ox-LDL-disposed endothelial cells, and its silencing hindered ox-LDL-triggered cell apoptosis and inflammation in endothelial cells. Consistently, we revealed that HDAC9 was prominently up-regulated in ox-LDL-disposed HUVECs. Moreover, miR-942-5p targeted HDAC9 to reduce ox-LDL-resulted HUVECs injury. Furthermore, we disclosed that circ_0003204 could modulate HDAC9 expression through decoying miR-942-5p.

In conclusion, circ_0003204 increased the expression of HDAC9 via sponging miR-942-5p, thereby preventing cell growth and promoting oxidative stress and inflammation in ox-LDL-mediated HUVECs. These findings demonstrated that circ_0003204 might be a promising therapeutic target for AS. The limitation of this work is the lack of *in vivo* experiments to verify the results of this research. In addition, more in-depth molecular mechanisms and their crosstalk with other studies need to be explored in future studies.

## Data Availability Statement

The raw data supporting the conclusions of this article will be made available by the authors, without undue reservation.

## Ethics Statement

The studies involving human participants were reviewed and approved by The First Affiliated Hospital of University of South China. The patients/participants provided their written informed consent to participate in this study.

## Author Contributions

HW: had full access to all of the data in the study and takes responsibility for the integrity of the data and the accuracy of the data analysis. HW and TY: study concept and design. TY and WL: acquisition of data. HW: critical revision of the manuscript for important intellectual content and study supervision. HW, TY, and WL: administrative, technical or material support. All authors: contributed to the article and approved the submitted version.

## Conflict of Interest

The authors declare that the research was conducted in the absence of any commercial or financial relationships that could be construed as a potential conflict of interest.

## Supplementary Material

Supplementary Figure 1 The expression levels of 6 candidate genes that might bind to miR-942-5p in HUVECs transfected with inhibitor NC or miR-942-5p inhibitor were detected by qPCR. Data were presented as mean ± SD, *n* = 3. ^*^*P* < 0.05, and ^**^*P* < 0.01.

Supplementary Figure 2 HUVECs were subjected to different transfections and treated with ox-LDL, and Bcl-2 protein level was examined using western blot. Data were presented as mean ± SD, *n* = 3. ^*^*P* < 0.05, and ^**^*P* < 0.01.

Supplementary Figure 3 After different transfections, the transcript levels of proliferation-related protein (PCNA) and apoptosis-related proteins (Bax and Bcl-2) were detected using qRT-PCR in ox-LDL-treated HUVECs. Data were presented as mean ± SD, *n* = 3. ^*^*P* < 0.05, ^**^*P* < 0.01, and ^***^*P* < 0.001.

Supplementary Figure 4 HDAC9 mRNA level was detected via qRT-PCR after different treatments. Data were presented as mean ± SD, *n* = 3. ^*^*P* < 0.05, ^**^*P* < 0.01, and ^***^*P* < 0.001.
