# Supplementary figures and images for: circ_0003204 Regulates Cell Growth, Oxidative Stress, and Inflammation in ox-LDL-Induced Vascular Endothelial Cells via Regulating miR-942-5p/HDAC9 Axis
Source: Front Cardiovasc Med. 2021 Apr 1;8:646832. doi: 10.3389/fcvm.2021.646832 (PMC8047481; doi:10.3389/fcvm.2021.646832)

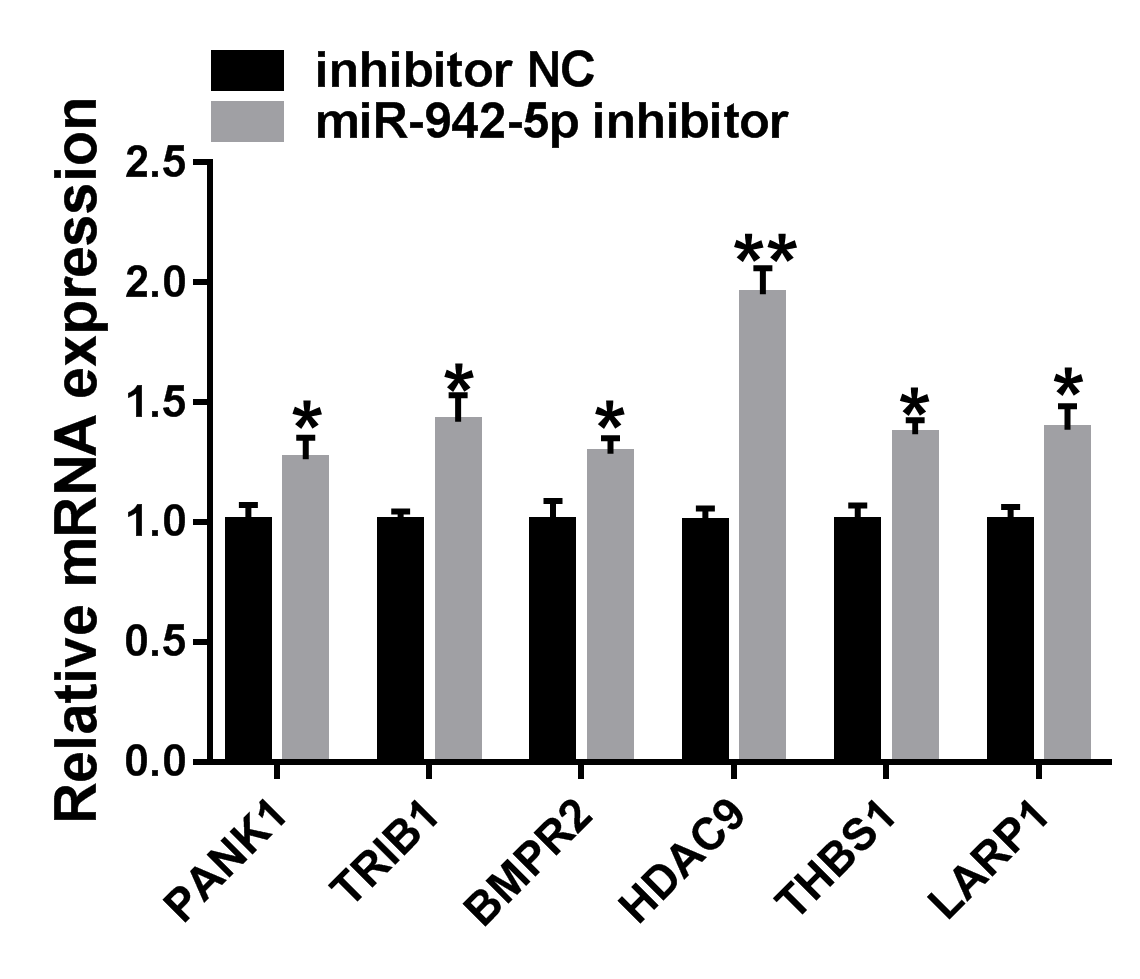

Supplement: Supplementary Figure 1 — The expression levels of 6 candidate genes that might bind to miR-942-5p in HUVECs transfected with inhibitor NC or miR-942-5p inhibitor were detected by qPCR. Data were presented as mean ± SD, n = 3. *P < 0.05, and **P < 0.01. [file Image_1.TIF]

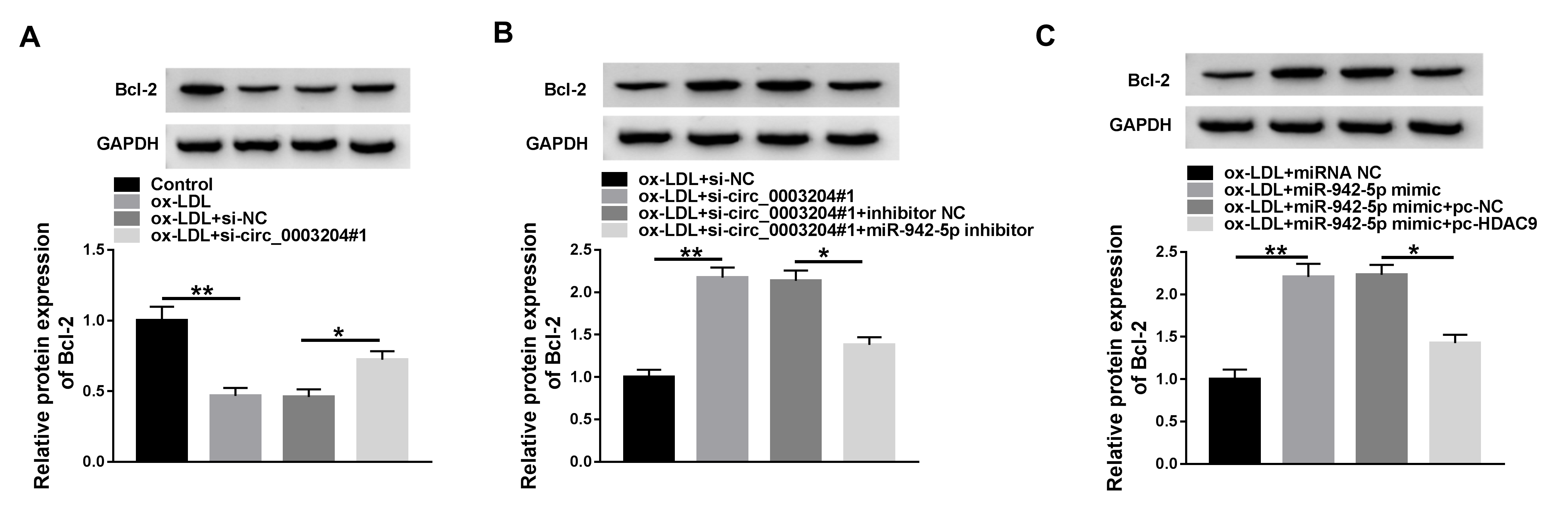

Supplement: Supplementary Figure 2 — HUVECs were subjected to different transfections and treated with ox-LDL, and Bcl-2 protein level was examined using western blot. Data were presented as mean ± SD, n = 3. *P < 0.05, and **P < 0.01. [file Image_2.TIF]

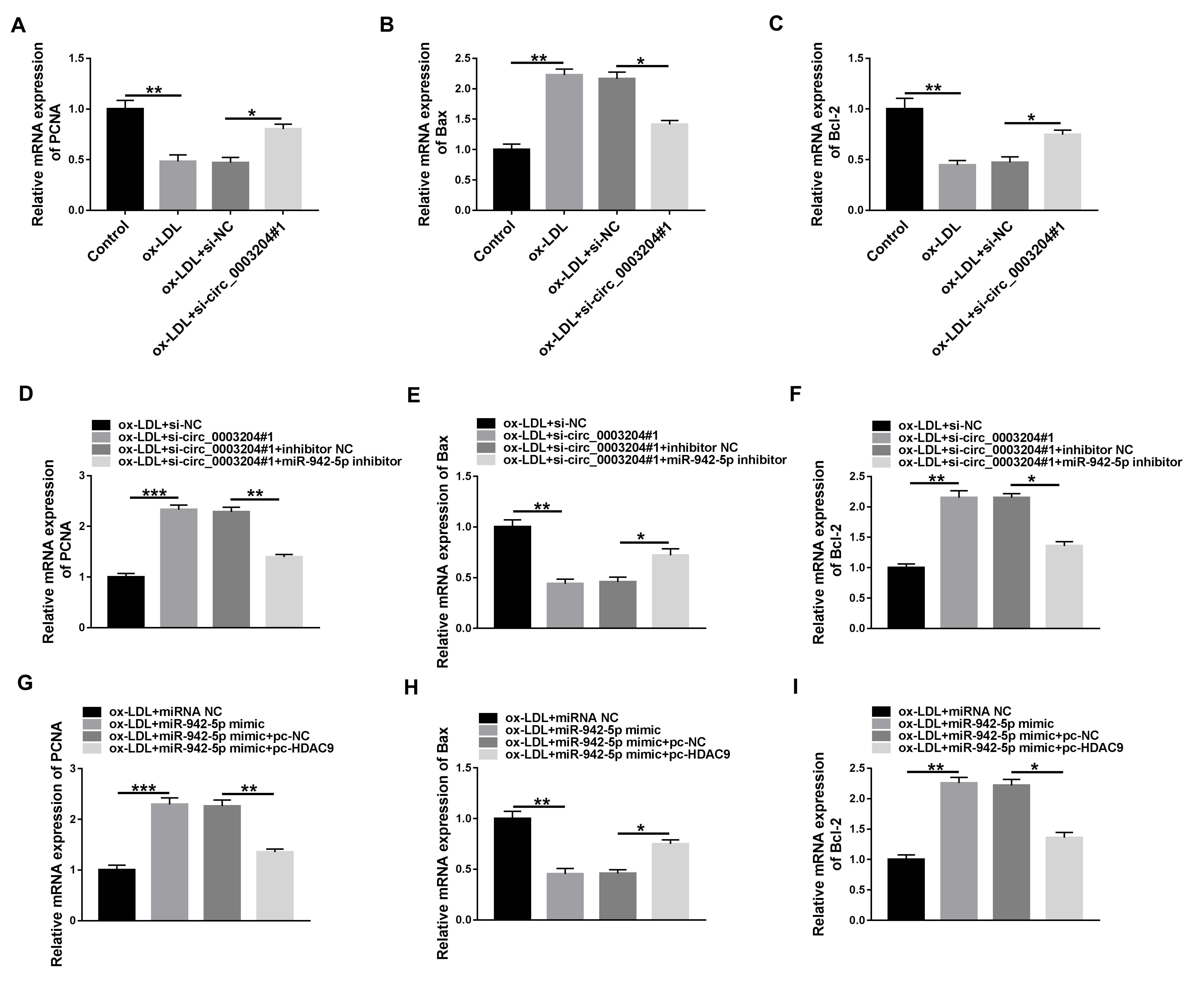

Supplement: Supplementary Figure 3 — After different transfections, the transcript levels of proliferation-related protein (PCNA) and apoptosis-related proteins (Bax and Bcl-2) were detected using qRT-PCR in ox-LDL-treated HUVECs. Data were presented as mean ± SD, n = 3. *P < 0.05, **P < 0.01, and ***P < 0.001. [file Image_3.TIF]

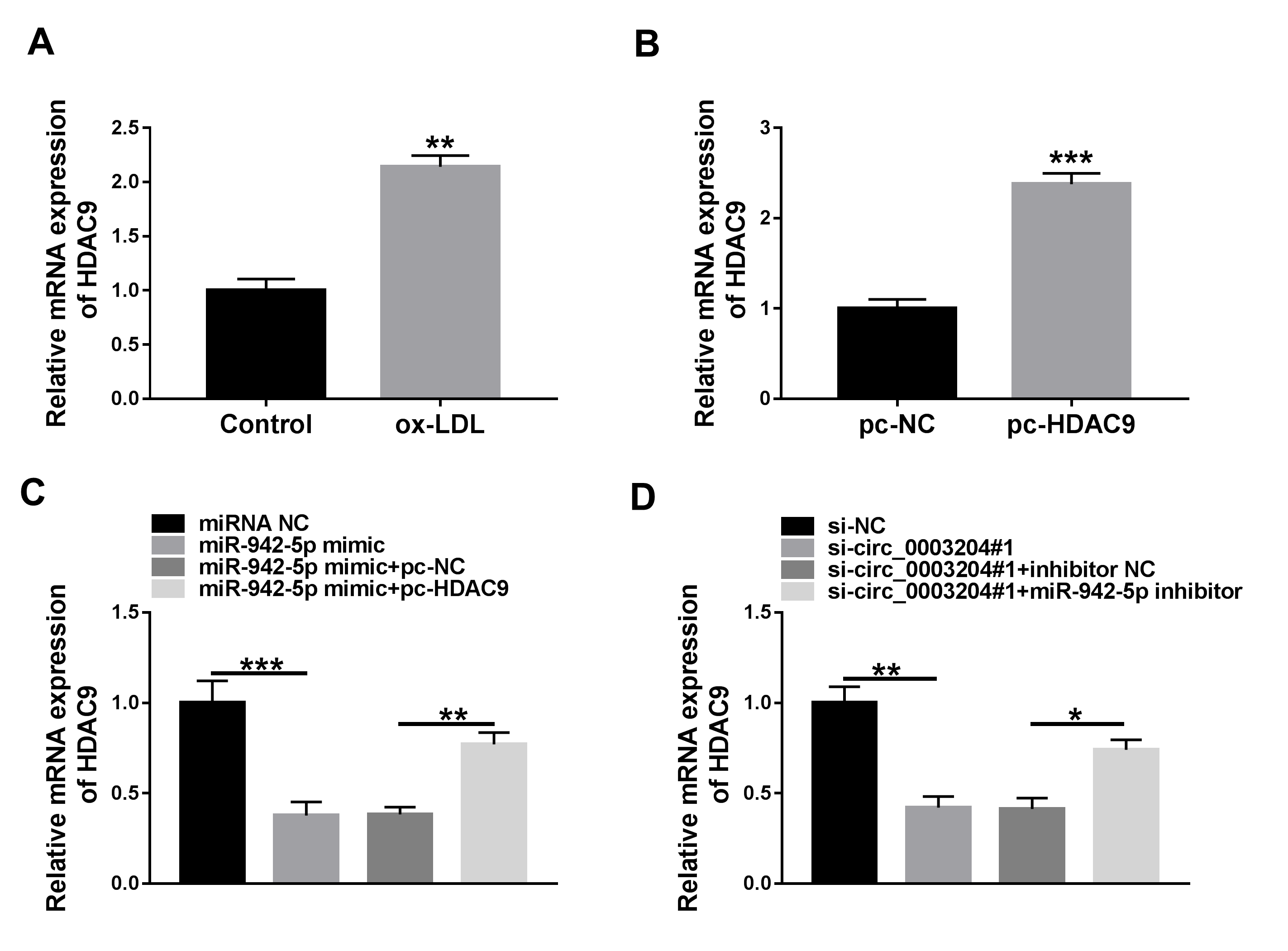

Supplement: Supplementary Figure 4 — HDAC9 mRNA level was detected via qRT-PCR after different treatments. Data were presented as mean ± SD, n = 3. *P < 0.05, **P < 0.01, and ***P < 0.001. [file Image_4.TIF]
